# A mechanosensory defect in a *C. elegans* amyloid-beta glutamatergic neuron model is reversed following exposure to *Salvia* species extracts

**DOI:** 10.17912/micropub.biology.000780

**Published:** 2023-03-20

**Authors:** Melissa L Schlein, Zekiye Ceren Arituluk, Cayman A Stephen, Guy A Caldwell, Lukasz Ciesla, Kim A Caldwell

**Affiliations:** 1 Biological Sciences, The University of Alabama, Tuscaloosa Alabama USA

## Abstract

Previous research has described promising neuroprotective and/or antioxidant properties for extracts derived from a few *Salvia* (sage) species. Here, six new *Salvia* species were isolated during flowering times from plants native to Turkey. Extracts were prepared and then examined for their potential to rescue both anterior and posterior mechanosensory behavioral defects in a transgenic *C. elegans* Alzheimer’s disease model that expresses human amyloid-beta (Aβ) peptide (1-42) exclusively in the glutamatergic neurons. Extracts from all six *Salvia* species rescued anterior touch response defects while only three rescued posterior touch response defects, compared to the Aβ controls.

**Figure 1.**
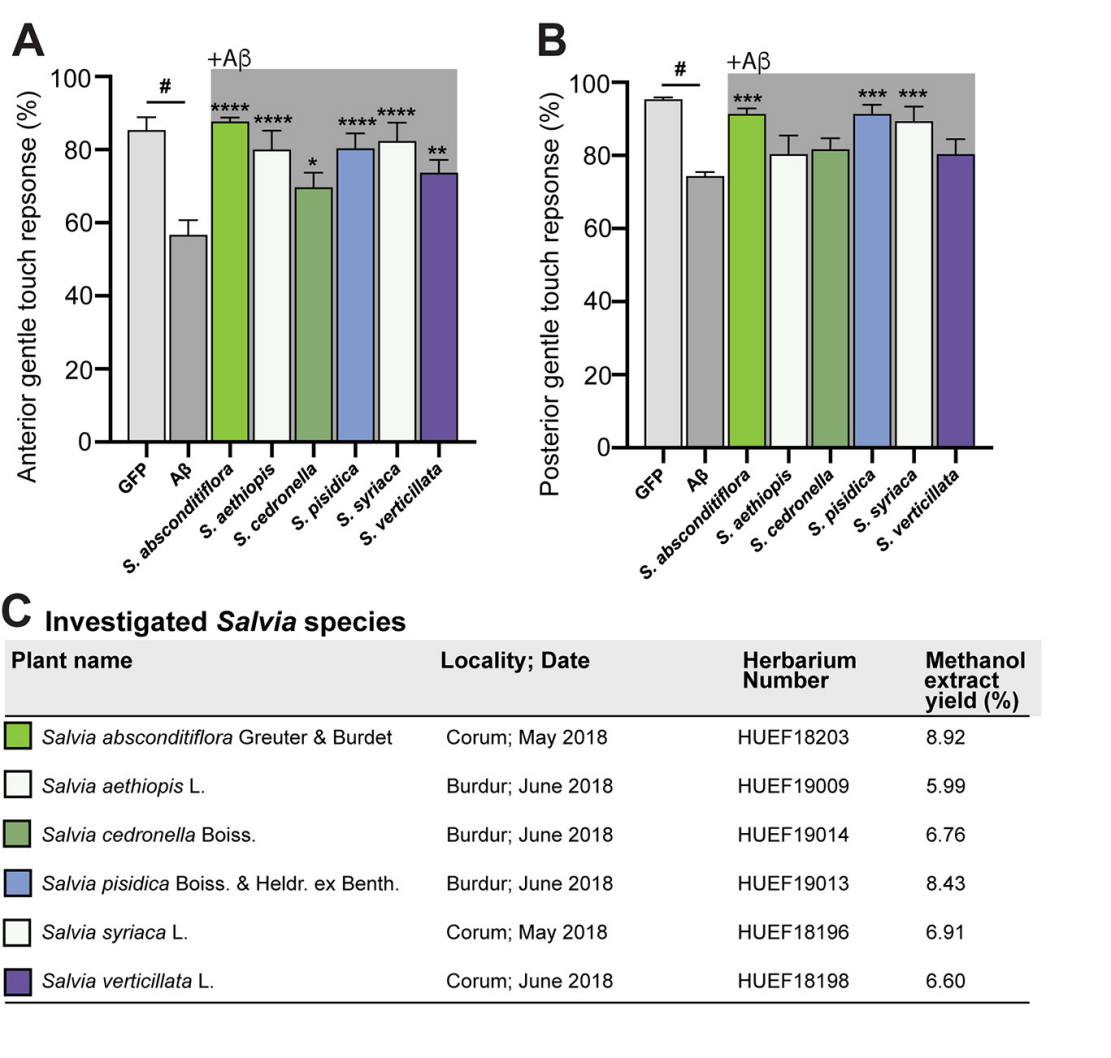
Comparing gentle touch response sensitivity in Aβ-expressing glutamatergic neurons. Control animals expressing only GFP in the glutamatergic neurons (light grey) vs. Aβ-expressing worms (dark grey) display significantly different gentle touch behavior in response to only solvent in both anterior (A) and posterior (B) assays. Gentle touch response is rescued in Aβ worms following exposure to several specific *Salvia* species extracts (A, B; shaded backgrounds). Error bars: s.e.m. N = 3; n = 30 per extract tested; one-way ANOVA with a Tukey’s post-hoc test, #P<0.0001; ***P<0.0005; *P<0.05; **P<0.005; ****P<0.0001. (C) Investigated *Salvia* species are displayed, along with the location in Turkey and date they were isolated. The Herbarium of Hacettepe University Faculty of Pharmacy number assigned to each of these isolates is provided, along with the yield of powdered, aerial air-dried plant material, initially extracted in methanol.

## Description

We created a transgenic nematode model of glutamatergic neurodegeneration by expressing the human amyloid-beta (Aβ) peptide (1-42) under a glutamatergic-specific promoter ( *eat-4* ). These animals display neurodegeneration and mechanosensory behavioral defects (Treusch et al., 2011; Griffin et al., 2018; Griffin et al., 2019) . These features are reflective of the glutamatergic hyperexcitability associated with the early stages of degeneration observed in AD patients (Palop and Mucke, 2009; Palop and Mucke, 2016) . In *C. elegans* , there are six sensory neurons that respond to gentle mechanosensory touch (Chalfie and Sulston, 1981) . Here, we asked if the mechanosensory defects associated with Aβ expression in the glutamatergic neurons could be pharmacologically rescued.

*Salvia* (sage) species have been shown to be a rich source of flavonoids, tannins, phenolic acids, and anthocyanins, and to exhibit antioxidant and/or neuroprotective activities (Mervić et al., 2021). Six species of sage were collected from flowering plants in different regions in Turkey ( Figure 1C ). The plants were dried, and extracts prepared, as detailed in the Methods.

We used assays for anterior and posterior gentle touch response to assess neuronal health in the presence of Aβ expression in the glutamatergic neurons of *C. elegans* . As shown in Figures 1A and 1B, controls exposed to 0.05% DMSO solvent only displayed significantly aberrant response to both anterior and posterior gentle touch in the P *_eat-4_* ::Aβ + P *_eat-4_* ::GFP(“Aβ”) animals vs. P *_eat-4_* ::GFP (“GFP”) worms lacking Aβ expression (Table 1). When Aβ worms were exposed to various sage extracts, all extracts significantly rescued the anterior touch response compared to solvent control ( Figure 1A ). When these same extracts were evaluated in Aβ worms for posterior touch response, three of six significantly rescued posterior touch response ( Figure 1B ).

Taken together, *S. absconditiflora* , *S. pisidica* and *S. syriaca* extracts provided the most significant benefit to transgenic *C. elegans* with Aβ expression in glutamatergic neurons, demonstrating rescue of mechanosensory deficit in both anterior and posterior touch response assays, whereas *S. aethiopis* , *S. cedronella,* and *S. verticillata* extracts improved only anterior touch response. We can only speculate why we observed a differential response between anterior and posterior touch response for some extracts. It is possible that there might be differentially regulated gene targets in the anterior vs. posterior touch neurons associated with sensitivity to certain sage extracts that accounts for rescue. For example, it was shown that a *Salvia miltiorrhiza* extract can significantly block Aβ-induced Ca ^2+^ intake in PC-12 cells (Zhou et al., 2011) . Additionally, solvent-treated Aβ control animals appeared to demonstrate a lower threshold of baseline sensitivity than was observed for posterior touch. This could imply that there are differences in the onset of behavioral dysfunction between the different touch neurons that could be revealed through multiple time-courses of testing. Future work could also include identification of the neuroprotective component(s) of the mixed extracts using *C. elegans* as a whole animal model for bioassay screening.

## Methods

**Plant materials** . Plant materials were collected in flowering times from different regions of Turkey. *Salvia absconditiflora* , *S. syriaca* , and *S. verticillata* were collected from Çorum province; *S. aethiopis* , *S. cedronella* , and *S. pisidica* were collected from Burdur province in 2018. Voucher specimens have been deposited in the Herbarium of Hacettepe University Faculty of Pharmacy (Ankara, Turkey), under related HUEF codes. The list of the investigated *Salvia* species with their HUEF codes is given Figure 1C . To prepare extracts, the air-dried and powdered aerial parts (20 g) of each plant was extracted three times with room temperature 200 mL methanol and then filtered. Individual plant filtrates were then combined and the methanol was evaporated using a rotary evaporator at > 40 °C and lyophilized. The percentage yields of methanolic extracts are given in Figure 1C . The methanolic extracts (1 g) were separately dissolved in 100 mL deionized water and partitioned by successive solvent extraction with *n-* hexane (3 x 100 mL), and dichloromethane (3 x 100 mL), respectively. The remaining aqueous phases were evaporated and lyophilized to yield crude extracts, which were each individually weighed before resuspension in 1 mL DMSO. Extracts were incorporated directly into the NGM media at a final concentration of 5 mg/Petri dish for each *Salvia* species tested in the *C. elegans* behavioral assays performed in each Petri dish.

***Salvia* worm exposure** . NGM plates containing *Salvia* at a final concentration of 5 mg were then seeded with *E. coli* OP50 and dried for 30 minutes in a sterile hood with the lids cracked open, before use. Three plates per *Salvia* per strain were prepared. Parental animals were grown on these plates and, 48 hours later, eggs were laid onto plates for 3-4 hours and the adults removed. The progeny were continuously exposed to the extracts at 20°C until day 4 post-hatching. For each worm strain, 30 adult animals were then examined for mechanosensory activity for each condition tested. Controls were exposed to DMSO only, at a final concentration of 0.05%.

**Mechanosensation assays** . Assays were performed as previously described (Chalfie and Sulston, 1981; Chalfie et al., 1985) . Sensitivity to gentle touch in *C. elegans* was assayed by gently stroking the same hermaphrodite animals at the anterior (posterior to the nose, but not at the nose) or posterior (just anterior to the anus) with an eyelash hair glued to the end of a Pasteur pipette. A positive response for anterior gentle touch was recorded if an animal ceased forward locomotion or began moving backward upon being stroked with an eyelash. Similarly, a positive result for posterior gentle touch was recorded if the animal ceased backward locomotion or began moving forward upon being stroked. This process was repeated 5 times per animal for each assay, and the number of positive responses to anterior and posterior gentle touch out of 5 was recorded. A total of 30 worms per strain were scored *per* biological replicate, with N = 3; n = 30 per strain tested; data represent the average of all three biological replicates with standard error of the mean (s.e.m.) calculated using GraphPad Prism (v. 8.0), as previously reported (Griffin et al., 2019) .

## Reagents

*Salvia* species ( Figure 1C )

*C. elegans* strains (Table 1)

methanol >99.8% ACS (BDH1135, VWR)

diatomaceous earth A (062819, Thermo Scientific)

dimethyl sulfoxide (D8418, Sigma-Aldrich)

Dionex ASE 150 solvent extractor

rotary evaporator

NGM agar plates

*E. coli* strain OP50 (saturated culture, previously grown in LB and stored at 4°C)

**Table 1.** *C. elegans* strains used in this work.

**Table:** 

STRAIN	GENOTYPE	SOURCE
UA198	*baIn34* [P *_eat-4_* ::Aβ, P *_myo-2_* ::mCherry]; *adIs1240* [P *_eat-4_* ::GFP]	Caldwell Lab
DA1240	*adIs1240* [P *_eat-4_* ::GFP] *lin-15B & lin-15A ( n765 ) *	CGC
